# Temporary antimetabolite treatment hold boosts SARS-CoV-2 vaccination–specific humoral and cellular immunity in kidney transplant recipients

**DOI:** 10.1172/jci.insight.157836

**Published:** 2022-05-09

**Authors:** Eva Schrezenmeier, Hector Rincon-Arevalo, Annika Jens, Ana-Luisa Stefanski, Charlotte Hammett, Bilgin Osmanodja, Nadine Koch, Bianca Zukunft, Julia Beck, Michael Oellerich, Vanessa Proß, Carolin Stahl, Mira Choi, Friederike Bachmann, Lutz Liefeldt, Petra Glander, Ekkehard Schütz, Kirsten Bornemann-Kolatzki, Covadonga López del Moral, Hubert Schrezenmeier, Carolin Ludwig, Bernd Jahrsdörfer, Kai-Uwe Eckardt, Nils Lachmann, Katja Kotsch, Thomas Dörner, Fabian Halleck, Arne Sattler, Klemens Budde

**Affiliations:** 1 Department of Nephrology and Medical Intensive Care and; 2 Department of Rheumatology and Clinical Immunology, Charité–Universitätsmedizin Berlin, corporate member of Freie Universität Berlin and Humboldt-Universität zu Berlin, Berlin, Germany.; 3 BIH Charité Clinician Scientist Program, BIH Biomedical Innovation Academy, Berlin Institute of Health at Charité–Universitätsmedizin Berlin, Berlin, Germany.; 4 German Rheumatism Research Centre Berlin (DRFZ), Berlin, Germany.; 5 Cellular Immunology and Immunogenetics Group, Faculty of Medicine, Institute of Medical Research, University of Antioquia (UdeA), Medellín, Colombia.; 6 Department of Clinical Pharmacology, Universitätsmedizin Göttingen, Göttingen, Germany.; 7 Chronix Biomedical GmbH, Göttingen, Germany.; 8 Department for General and Visceral Surgery, Charité–Universitätsmedizin Berlin, corporate member of Freie Universität Berlin and Humboldt-Universität zu Berlin, Berlin, Germany.; 9 Institute of Transfusion Medicine, Ulm University, Ulm, Germany.; 10 Institute for Clinical Transfusion Medicine and Immunogenetics, German Red Cross Blood Transfusion Service Baden-Württemberg–Hessen and University Hospital Ulm, Ulm, Germany.; 11 Center for Tumor Medicine, H&I Laboratory, Charité–Universitätsmedizin Berlin, corporate member of Freie Universität Berlin and Humboldt-Universität zu Berlin, Berlin, Germany.

**Keywords:** COVID-19, Vaccines, Organ transplantation

## Abstract

Transplant recipients exhibit an impaired protective immunity after SARS-CoV-2 vaccination, potentially caused by mycophenolate (MPA) immunosuppression. Recent data from patients with autoimmune disorders suggest that temporary MPA hold might greatly improve booster vaccination outcomes. We applied a fourth dose of SARS-CoV-2 vaccine to 29 kidney transplant recipients during a temporary (5 weeks) MPA/azathioprine hold, who had not mounted a humoral immune response to previous vaccinations. Seroconversion until day 32 after vaccination was observed in 76% of patients, associated with acquisition of virus-neutralizing capacity. Interestingly, 21/25 (84%) calcineurin inhibitor–treated patients responded, but only 1/4 belatacept-treated patients responded. In line with humoral responses, counts and relative frequencies of spike receptor binding domain–specific (RBD-specific) B cells were markedly increased on day 7 after vaccination, with an increase in RBD-specific CD27^++^CD38^+^ plasmablasts. Whereas overall proportions of spike-reactive CD4^+^ T cells remained unaltered after the fourth dose, frequencies were positively correlated with specific IgG levels. Importantly, antigen-specific proliferating Ki67^+^ and in vivo–activated programmed cell death 1–positive T cells significantly increased after revaccination during MPA hold, whereas cytokine production and memory differentiation remained unaffected. In summary, antimetabolite hold augmented all arms of immunity during booster vaccination. These data suggest further studies of antimetabolite hold in kidney transplant recipients.

## Graphical abstract

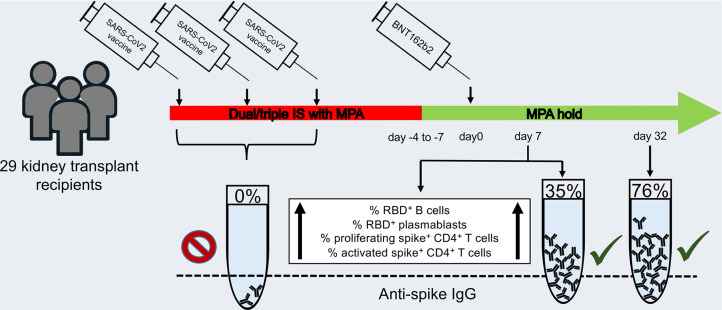

## Introduction

Protection of kidney transplant recipients (KTRs) from COVID-19, caused by SARS-CoV-2, has not been sufficiently achieved by conventional vaccination protocols. Mortality of fully vaccinated KTRs after infection remains unacceptably high, with almost 8% mortality in a registry analysis from the United Kingdom (1) and up to 20% in other cohorts (2, 3), despite the presence of vaccine-specific T cells. Our previous studies revealed a strong impairment of both humoral and cellular immunity in transplant recipients after 2 doses of BNT162b2, with antigen-specific B and T cell responses being both quantitatively and functionally affected (4, 5). Since the majority of KTRs do not benefit from a third dose (6), modified vaccination protocols are required to achieve protection of this at-risk population.

Analysis of large transplant patient cohorts indicated that mycophenolate-based (MPA-based) treatment constitutes a major risk factor for impairment of vaccine-induced humoral immunity (7, 8). In line with the aforementioned finding, a case series with patients with rheumatic and musculoskeletal diseases demonstrated that temporary hold of MPA leads to augmented humoral responses to SARS-CoV-2 vaccination (9). According to European guidelines, the majority of kidney-transplanted individuals receive triple immunosuppressive medication including calcineurin inhibitors (CNIs), corticosteroids (CS), and MPA. Withdrawal of steroids or MPA in a tacrolimus-based treatment protocol for up to 3 years has been shown to be safe in a large multicenter study, with no increase in acute rejections or impaired kidney function (10). Similar data were obtained from other trials (11–13). Furthermore, hold of MPA is routinely recommended during pregnancy (14), underlining the feasibility of this approach. To examine the impact of short-term MPA withdrawal on vaccination outcome, 29 KTRs, being seronegative after triple SARS-CoV-2 vaccination, were converted to an MPA-free immunosuppressive regimen. Patients were closely monitored for clinical parameters, including kidney function, anti-HLA antibodies, and donor-derived cell-free DNA (dd-cfDNA) (15, 16); assessment of vaccine-specific immunity encompassed in-depth analysis of specific B and T cell analyses, IgG and IgA levels, and neutralization capacity.

## Results

### Vaccination-induced humoral and B cell immunity.

The study cohort included 29 KTRs with a lack of serological response after a 3-dose vaccine protocol. Fourteen patients were homogeneously vaccinated (3 times with mRNA vaccine); 15 patients were vaccinated heterologously (mixed mRNA and vector based). All patients received BNT162b2 (BioNTech/Pfizer) as a fourth vaccine. Mean time interval between the third and fourth vaccinations was 59.1 (±12.6) days. All patients were initially on antimetabolite treatment, 28/29 on MPA and 1/29 on azathioprine (Aza). Among the 29 KTRs, 26 received CNI-based medication while 4 patients received belatacept. All patients stopped MPA or Aza 4–7 days before the fourth vaccination, based on the assumption that pharmacodynamic drug effects wane after 3–4 days (17). Treatment was paused until days 28–35 (mean depicted as “day 32” in all figures; second time point for serological response analysis). In patients with no or few CS, CS were restarted or increased to 5 mg prednisone equivalent together with MPA hold. In the 4 patients on belatacept, 2 stopped MPA and 1 Aza, while it was replaced by CS in only 1 patient. One belatacept-treated patient was switched to tacrolimus and CS. Demographics are summarized in Table 1.

**Figure 1:**
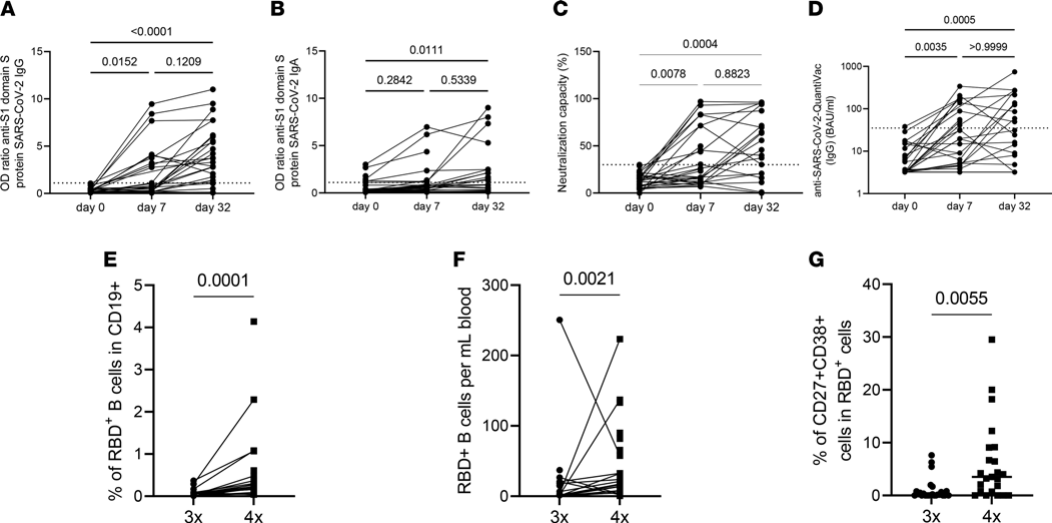
Humoral immune responses and specific B cell immunity after fourth vaccination in KTRs. Humoral vaccine-specific immune responses were assessed by ELISA for anti–spike protein S1 IgG (*n* = 29) (**A**), spike protein S1 IgA (*n* = 29 day 0, 7, *n* = 21 day 32) (**B**), and virus neutralization by a blocking ELISA (*n* = 29 day 0, 7, *n* = 21 day 32) (**C**) as well as by QuantiVac (IgG) (*n* = 29/29 day 0, 7, *n* = 21 day 32) (**D**) at the indicated time points in KTRs after administration of a fourth dose of BNT162b2. Thresholds defining a positive response are indicated by dotted lines. (**E**) Relative frequencies (3 times — *n* = 25, 4 times — *n* = 23) and (**F**) absolute counts (3 times — *n* = 23, 4 times — *n* = 23) of RBD-specific CD19^+^ B cells 7 ± 2 days after fourth vaccination with BNT162b2. (**G**) Frequency of RBD-specific CD27^++^CD38^+^ plasmablasts. (**A**–**D**) Kruskal-Wallis with Dunn’s posttest. (**E**–**G**) Mann-Whitney *U* test. Where applicable, graphs show means ± SD.

Seroconversion (OD ratio > 1.1) for anti–S1 domain IgG occurred in 10/29 (34.5%) individuals until day 7 after the fourth vaccination, while anti–S1 domain IgA was positive in 7/29 KTRs (24.1%). Neutralization capacity above 30% was achieved in 11/29 (37.9%) patients (Figure 1, A–C). On day 32 after vaccination, 22/29 (76%) patients showed anti–S1 domain IgG levels above the threshold for positivity. Anti–S1 domain IgA and neutralization capacity levels were unavailable for 8 individuals. For the remaining individuals, IgA was positive in 11/21 patients (52.2%), and neutralization capacity was above threshold in 15/21 (71.4%) patients (Figure 1, A–C). In patients with CNI treatment before vaccination, anti–S1 domain IgG seroconversion occurred in 21/25 (84%) patients, while 3/4 patients on belatacept remained negative; only 1 became weakly positive just above the threshold on day 32 (Supplemental Figure 1B; supplemental material available online with this article; https://doi.org/10.1172/jci.insight.157836DS1). Anti–S1 domain IgG on day 32 did not differ between patients upon heterologous or homologous vaccination (Supplemental Figure 1A). For comparability of the study results, an assay determining standardized binding antibody units (BAU) for anti–S1 domain IgG (QuantiVac) was also performed. All 10/29 individuals showing IgG levels above threshold on day 7 based on OD ratios were also positive in the QuantiVac assay (Figure 1D). Given that not all samples were available for the QuantiVac assay on day 32, a 1:1 comparison was not possible for all individuals. Here, we determined 12/21 (57.1%) to be above the lower limit of 35.2 BAU/mL (Figure 1D). Complete serological nonresponders, defined as IgG < 3.5 BAU/mL before fourth vaccination (*n* = 15), showed a trend toward lower titers at day 32 compared with partial nonresponders with IgG > 3.5 BAU/mL before fourth vaccination (Supplemental Figure 1D), but this difference did not reach statistical significance.

Antigen-specific B cells were identified by fluorescence double-labeling of reactive cells with recombinant receptor binding domain (RBD) (5) (Supplemental Figure 1E). Frequencies and absolute counts of RBD^+^ B cells increased 7 days after vaccination compared with baseline (Figure 1, E and F). Interestingly, the frequency of RBD^+^ plasmablasts, which have been shown to be an early sign of vaccine response (5), increased after 4 vaccinations (Figure 1G).

The IMPDH activity in erythrocytes has recently been described as a useful pharmacodynamic marker for MPA exposure that reflects the MPA exposure after 8 weeks of constant dosing (17). High IMPDH levels were found in patients with MPA toxicity and low levels in patients with biopsy-proven acute rejections (17). In the current cohort, the mean IMPDH activity before MPA hold at steady state was 1192.73 pmol XMP/h/mg Hb (±474.24), and IMPDH activity did not negatively correlate with anti–S1 domain IgG on day 32 (Supplemental Figure 1C).

### Vaccination-specific CD4^+^ T cell responses.

**Figure 2:**
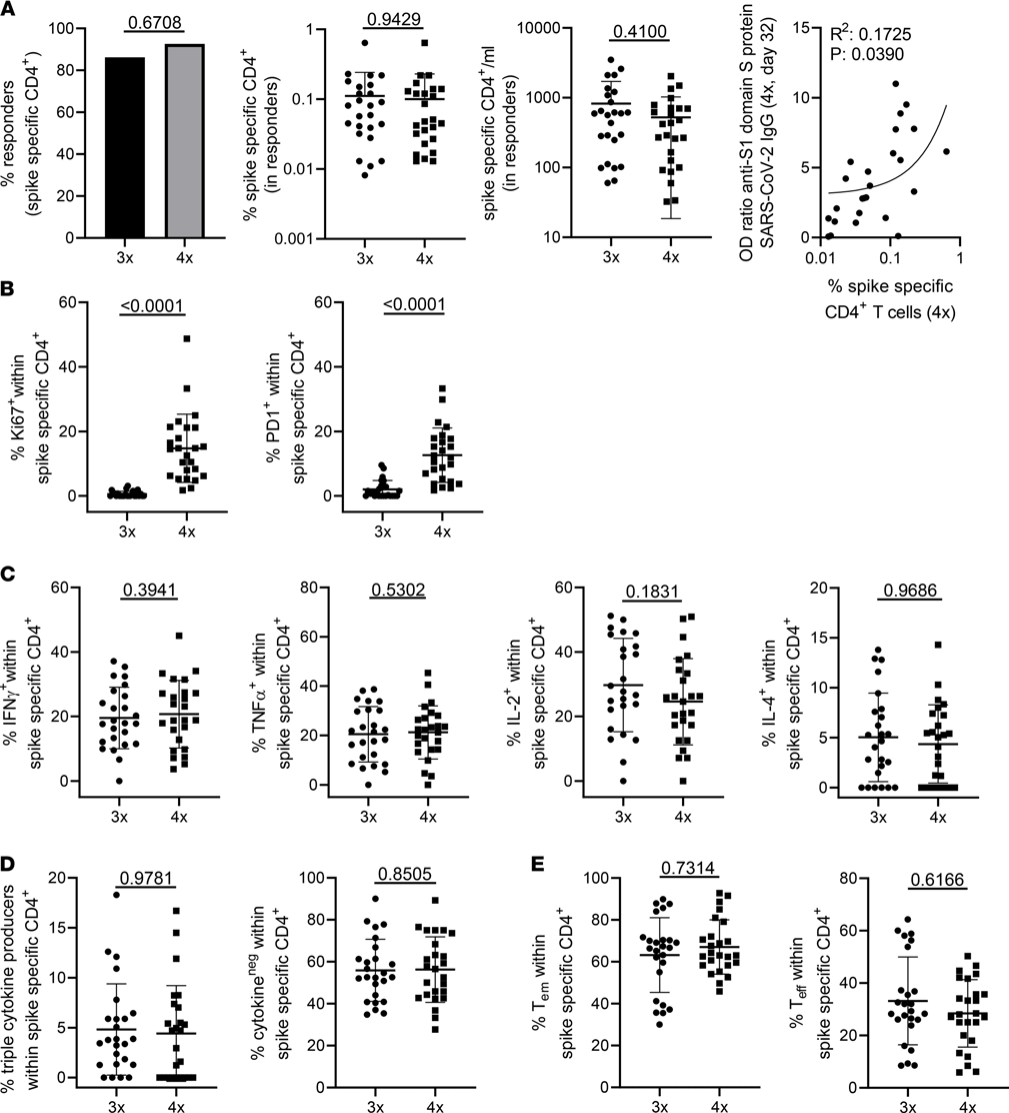
Assessment of T cell reactivity. PBMCs of KTRs were stimulated with spike peptide mix or left unstimulated. Specific CD4^+^ T cells were detected immediately before the fourth dose and 7 days thereafter by flow cytometry according to coexpression of CD154 and CD137. (**A**) The portion of individuals with a cellular response (left, Fisher’s exact test, 3 times — *n* = 29, 4 times — *n* = 27), relative (middle/left, paired Wilcoxon’s test) and absolute (middle/right, paired Wilcoxon’s test) frequencies of specific CD4^+^ T cells, and the correlation between relative frequencies and levels of anti–spike S1 domain IgG (right, simple linear regression). (**B**) Frequencies of antigen-reactive CD4^+^ T cells expressing Ki67 (left, paired Wilcoxon’s test) or PD-1 (right, paired Wilcoxon’s test). (**C**) Expression of IFN-γ (paired *t* test), TNF-α (paired *t* test), IL-2 (paired *t* test), and IL-4 (paired *t* test) in antigen-specific T cells. (**D**) Analysis of IFN-γ^+^TNF-α^+^IL-2^+^ “triple^+^” polyfunctional (paired Wilcoxon’s test, left) and non-cytokine-producing cells (paired Wilcoxon’s test, right). (**E**) Memory/effector subset differentiation of antigen-specific CD4^+^ T cells (T_EM_: effector memory [left, paired Wilcoxon’s test], T_eff_: effector [right, paired Wilcoxon’s test]). In all analyses except in responder rate calculation (**A**), *n* = 25 individuals were included per group. Where applicable, graphs show means ± SD.

SARS-CoV-2 spike protein–reactive CD4^+^ T helper cells were detected within PBMCs based on activation-induced coexpression of CD154 and CD137 after stimulation with 15-mer peptides (overlapping by 11 amino acids, respectively) covering the complete spike glycoprotein sequence, as previously reported (4, 18). The gating strategy, including subset identification, is depicted in Supplemental Figure 2. A positive T cell response was defined when stimulated PBMCs contained more than 3-fold higher frequencies of CD154^+^CD137^+^CD4^+^ T cells as compared with the unstimulated control (stimulation index of 3) with at least 20 events, being in accordance with comparable studies (19). The prevalence of cellular responders was similar (>85%) after the third and fourth vaccinations, with no significant differences in relative and absolute frequencies of antigen-reactive T cells. Of note, levels of anti–SARS-CoV-2 spike S1 domain–specific IgG were positively correlated with frequencies of spike-specific T cells (Figure 2A).

Spike-specific T cells of individuals after the fourth vaccination contained significantly higher proportions of cells expressing the proliferation marker Ki67; the same applied to expression of programmed cell death 1 (PD-1), indicating recent in vivo activation (Figure 2B). Interestingly, we did not detect significantly elevated frequencies of antigen-specific T cells expressing IFN-γ, TNF-α, IL-2, or IL-4 after the fourth dose (Figure 2C); this also applied to proportions of specific polyfunctional IFN-γ^+^TNF-α^+^IL-2^+^ T cells or cells secreting none of the 3 cytokines (Figure 2D). IL-4 was excluded from polyfunctionality analyses due to low frequencies of positive cells. Antigen-reactive T cells from individuals after the third and fourth doses showed similar frequencies of CD45RO^+^CD62L^–^ effector memory and CD45RO^–^CD62L^–^ effector T cells, respectively (Figure 2E). We further compared frequencies of Ki67 and PD-1 with a matched past cohort after third vaccination where 24/25 patients received standard immunosuppressive medication including MPA (20); demographics are summarized in Supplemental Table 1. We found that frequencies of Ki67^+^ spike-specific CD4^+^ cells were significantly higher 7 days after vaccination in antimetabolite-free patients as compared with the control cohort receiving MPA. This observation did not apply to proportions of PD-1^+^ cells (Supplemental Figure 2B).

### HLA antibody testing and dd-cfDNA.

Since conversion of an established immunosuppressive regimen bears the risk of adverse events such as rejection or the generation of de novo HLA antibodies, we performed anti-HLA antibody testing before and after vaccination in 27/29 study participants. No patient developed de novo HLA antibodies, and antibody pattern and strength remained unchanged in 3 patients with preexisting donor-specific HLA antibodies before vaccination. Kidney function remained stable after day 32. A novel marker for subclinical allograft injury and rejection, dd-cfDNA (16, 21), was available for 16/29 KTRs and did not increase.

## Discussion

The current study investigates the immunological impact of a fourth dose of a SARS-CoV-2 vaccine during short-term MPA hold in KTRs who did not seroconvert after 3 vaccine doses. We found a significant increase in humoral responders at day 32 (76% irrespective of previous treatment, 84% in individuals on standard CNI regimen), with an increase in neutralizing antibodies and occurrences of vaccine-specific B cells and plasmablasts. We further observed higher ex vivo activation of spike-specific T cells that quantitatively correlated with spike S1–specific IgG at day 32.

So far, the response to SARS-CoV-2 mRNA- and vector-based vaccines in KTRs has been disappointing (4, 22), resulting in high infection and hospitalization rates in fully vaccinated individuals (3). The early recommendation of a third vaccination for solid organ recipients, which has since been extended to the general population (23), entailed IgG seroconversion rates of up to 68% (24), a feature that was, however, not reproducible for patients receiving triple immunosuppressive therapy and being seronegative before the third vaccination (6). Positive but low prevaccination IgG levels are associated with superior outcomes after a fourth dose on stable immunosuppression, leading to seroconversion rates of 42%–50% (25, 26). Still, seronegative patients after repeated revaccination clearly represent the most vulnerable subgroup with higher risk of severe COVID-19 (1, 3). This is becoming increasingly important with the emergence of new viral variants, resulting in reduced neutralization capacity even in healthy individuals (27).

Our approach to withdraw MPA, followed by revaccination, obviously affected all arms of immunity with a strong impact on B cell activation and differentiation, thereby boosting spike-specific antibody production. Interestingly, seroconversion was already detectable on day 7 in 34.4% of patients, as compared with only 12% of individuals receiving a third dose under MPA treatment (6), highlighting enhanced immune kinetics in the absence of antimetabolites. Importantly, seroconversion did not depend on the type of previous vaccines since we did not observe differences between patients who received a heterologous or homologous vaccination regimen.

So far, antimetabolites including MPA and Aza have been primarily demonstrated to impair B cell proliferation and plasmablast formation in autoimmunity (28) but to also block expansion and activation of naive and memory B cells isolated from healthy individuals (29–31). Mechanistically, MPA inhibits IL-6–mediated STAT3 signaling, a prerequisite for plasma cells’ differentiation (32) and critical for their survival and immunoglobulin secretion in the bone marrow (33). To the best of our knowledge, our data, for the first time, verify MPA’s effects on B cells in an antigen-specific context, including impairment of spike-specific CD27^++^CD38^+^ plasmablast formation. With respect to the T cell compartment, our data are in line with recent studies showing that production of IFN-γ, TNF-α, or IL-2 by polyomavirus BK–specific memory T cells or bulk mucosal associated invariant T cells remains unaffected by MPA (34, 35). This might be related to the fact that the IL-6/STAT3 axis is selectively involved in differentiation of IL-17–secreting Th17 cells (36). As opposed to cytokine production, in vivo activation, as evidenced by Ki67 and PD-1 expression, significantly increased in vaccine-specific CD4^+^ T cells after MPA hold, suggesting that initial activation/proliferation is more sensitive to antimetabolites, as has been demonstrated for purified naive human T cells (37). For Ki67, dependency on antimetabolite treatment is supported by our historical cohort data where expression was analyzed at the same time point in MPA-treated individuals. Interestingly, our finding that higher frequencies of spike-reactive T cells correlated with specific IgG levels mirrors early analyses of healthy SARS-CoV-2 vaccinees (38).

As an obvious limitation of our approach, a subgroup of patients with previous or ongoing belatacept treatment did not benefit from MPA hold. Although our patient numbers were small, our data support the notion that belatacept efficiently inhibits vaccine responses (39) irrespective of the presence of antimetabolites, suggesting that other approaches are needed for belatacept-treated patients.

Further limitations of our study include that patients were at a median of 9.9 years after transplantation with stable graft function, thereby representing a very low risk group for an alloimmune response. In this context, an earlier time point after transplantation has been identified as a risk factor for a poor vaccine response in previous studies (22) while these patients may also be at a greater risk for rejection and anti-HLA antibody formation following temporary immunosuppression reduction. The transferability of our approach is further limited by the small sample size and the lack of a control group with continued antimetabolite treatment receiving a fourth vaccination. Safety data in our study have to be interpreted with caution due to short follow-up since anti-HLA antibody formation and deterioration of kidney function might develop over time. To address these aspects, larger cohorts with longer follow-up times are needed.

In summary, our data provide evidence that temporary hold of MPA for 5 weeks in patients under previous CNI, MPA, ±CS is a viable option to accelerate and increase vaccine efficacy in KTRs, particularly given that graft function remained stable and no rejection episodes or increases in anti-HLA antibodies and dd-cfDNA plasma concentrations were observed within the observation period. Our study thus highlights a potential rapid vaccination strategy for at-risk patients under standard CNI-based immunosuppression that warrants testing in larger cohorts.

## Methods

### Study protocol and participants.

Patient demographics are summarized in Table 1. Peripheral blood and serum samples were collected immediately before and 7 ± 2 days after the fourth vaccination (humoral, B and T cell analyses) and 28–35 days (mean “day 32”) after the fourth dose (humoral analyses, HLA antibodies, assessment of dd-cfDNA).

### Serological assessment.

Serological assessment was performed as previously reported (5, 6, 40). In brief, SARS-CoV-2 S1 domain–specific IgG and IgA were determined by ELISA (EUROIMMUN). Previous or current SARS-CoV-2 infection was excluded based on medical history in combination with negativity on a SARS-CoV-2 nucleoprotein–specific ELISA (EUROIMMUN). Samples were considered positive with OD ratios of ≥1.1 as per manufacturer’s guidelines. An OD ratio value was determined by calculating the ratio of the OD of the respective test sample over the OD of the internal calibrator provided with the ELISA kit. For determination of standardized BAU, the QuantiVac assay (EUROIMMUN) was used with values >35.2 BAU/mL considered positive according to the manufacturer’s guidelines. Virus neutralization capacity of sera was analyzed using a surrogate SARS-CoV-2 neutralization test (GenScript), with more than 30% being defined as a positive response as described previously (41, 42).

### Clinical parameters.

Clinical parameters were extracted from our patient database (43). The EGFR was calculated based on creatinine levels by the Chronic Kidney Disease Epidemiology Collaboration formula. Data were collected at a median of 18 days (range 12.5, 31.75) before fourth vaccination and at a median of 124 days (range 94, 139) after the fourth vaccination. Data for EGFR were available for both time points for 28/29 patients. Albumin/creatinine (mg/g) in the spontaneous urine was available for 24/29 patients for both time points. Monitoring of HLA antibodies was performed as described previously (44) together with the day 32 follow-up time point.

### Erythrocyte IMPDH measurement.

Erythrocyte IMPDH activity was measured as described recently (17) as a part of clinical practice. The last available IMPDH at steady state before change in MPA dose is reported in Table 1.

### Measurement of dd-cfDNA.

The measurement of dd-cfDNA was performed as described previously (15, 45) at day 32. In brief, for each patient, 4 informative independent single nucleotide polymorphism (SNP) assays were used, for which the recipient has a homozygous allelic state and the graft carries at least 1 heterozygous allele. These were selected from a predefined set of 40 SNPs. These 4 SNP assays were used to quantify the dd-cfDNA (%) concentration, defined as donor alleles/(donor alleles + recipient alleles). Results for SNPs with heterozygous graft genotypes were corrected by a factor 2. Total cfDNA was extracted from up to 8 mL of plasma collected in certified blood collection tubes (Streck Corp). The measurement was performed using droplet-digital PCR. Results were corrected for extraction efficiency and cfDNA fragmentation in absolute quantification, as described previously (21). The absolute concentration of dd-cfDNA per mL of plasma was calculated by multiplying total cfDNA (copies/mL) and dd-cfDNA (%). Time-dependent changes for total cfDNA and dd-cfDNA fraction (%) in the posttransplant course were assessed in a cohort of 300 KTRs, as described previously (46).

### Characterization of antigen-specific B and T cells.

All experiments were performed as previously described (4, 5, 18). In brief, PBMCs were isolated by density gradient centrifugation using Ficoll-Paque PLUS (GE Healthcare Bio-Sciences). B cells were detected within PBMCs by flow cytometry and gated as CD19^+^CD3^–^CD14^–^ among single live lymphocytes (gating strategy depicted in Supplemental Figure 1E). For flow cytometric analysis, the following fluorochrome-labeled antibodies were used: CD14 (M5E2, BD Biosciences [BD]), CD3 (UCHT1, BD), CD27 (L128, BD), CD19 (SJ25C1, BD), CD24 (ML5, BD), IgD (IA6-2, BioLegend), and CD38 (HIT2, BioLegend). Antigen-specific B cells were identified (Supplemental Figure 1E) by double-staining with recombinant purified RBD (DAGC149, Creative Diagnostics) conjugated to Alexa Fluor 647 or Alexa Fluor 488. For identification of vaccine-reactive T cells, 3 × 10^6^ to 5 × 10^6^ PBMCs were stimulated for 16 hours with overlapping 15-mers covering the complete SARS-CoV-2 spike protein (1 μg/mL per peptide; JPT). Specific CD4^+^ T helper cells were identified based on CD154 and CD137 coexpression as shown in Supplemental Figure 2. For labeling of surface markers, antibodies against CD3 (SK7, BioLegend), CD4 (SK3, BD), CD8 (SK1, eBioscience, Thermo Fisher Scientific), CD45RO (UCHL1, BioLegend), CD62L (DREG-56, BioLegend), and PD1 (EH12.1, Becton Dickinson) were used. A dump channel served to exclude unwanted cells containing CD14^+^ (M5E2, BioLegend), CD19^+^ (HIB19, BioLegend), and dead cells (fixable live/dead, BioLegend). After surface staining, cells were fixed in FACS Lysing Solution (Becton Dickinson), permeabilized in FACS Perm II Solution (Becton Dickinson), and stained intracellularly with anti-CD154 (24-31, BioLegend), anti-CD137 (4B4-1, BioLegend), anti–TNF-α (MAb11, BioLegend), anti–IFN-γ (4SB3, eBioscience, Thermo Fisher Scientific), anti–IL-2 (MQ1-17H12, BioLegend), anti-Ki67 (B56, Becton Dickinson), and anti–IL-4 (MP4-25D2, BioLegend). Data acquisition was performed using a BD LSRFortessa X-20.

### Statistics.

FACS data were analyzed with FlowJo 10 (BD). The gating strategies for analysis of antigen-reactive B and T cells are illustrated in Supplemental Figures 1 and 2. Coexpression of cytokines was quantified by Boolean gating in FlowJo. Statistical analysis and graph preparation were conducted in GraphPad Prism 8. Normal distribution of data was assessed using the Kolmogorov-Smirnov test. Depending on the presence of normal distribution, a 2-tailed *t* or Wilcoxon’s test was used for paired 2-group comparisons. For multiple comparisons, a 2-way ANOVA with Holm-Šidák posttest or Kruskal-Wallis test with Dunn’s posttest were chosen. For analysis of contingency tables, Fisher’s exact test was applied. *P* values less than 0.05 were considered statistically significant.

### Study approval.

All participants gave written informed consent for sample collection according to the approval of the ethics committees of the Charité–Universitätsmedizin Berlin (EA2/010/21, EA4/188/20).

## Author contributions

E Schrezenmeier, AS, FH, and KB designed the study and wrote the manuscript. HRA, ALS, E Schrezenmeier, AS, VP, and CS performed experiments. E Schrezenmeier, CH, AJ, KB, NK, BZ, FB, and MC recruited patients. LL and PG provided IMPDH data. KBK, E Schütz, BO, JB, and MO performed assessment of dd-cfDNA. NL and CLDM performed HLA antibody testing. HS, CL, and BJ were responsible for serological studies. E Schrezenmeier, KB, KK, TD, KUE, and FH supervised the work and provided funding. All authors read and approved the manuscript. Contributions of equally contributing authors were determined by consensus.

## Supplementary Material

Supplemental data

Supplemental table 1

## Figures and Tables

**Table 1:**
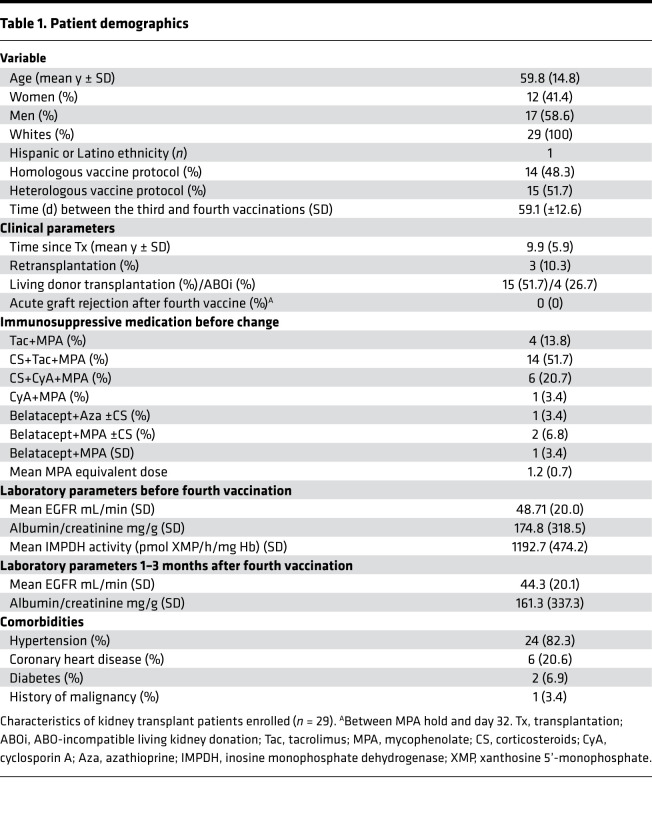
Patient demographics

## Version 1 03/29/2022

In-Press Preview

## Version 2 05/09/2022

Electronic publication
