# Supplementary material for: Temporary antimetabolite treatment hold boosts SARS-CoV-2 vaccination–specific humoral and cellular immunity in kidney transplant recipients
Source: JCI Insight. 2022 May 9;7(9):e157836. doi: 10.1172/jci.insight.157836 (PMC9090237; doi:10.1172/jci.insight.157836)
Supplement: Supplemental data [file jciinsight-7-157836-s060.pdf]

Supplemental Figure 1, Schrezenmeier et al.

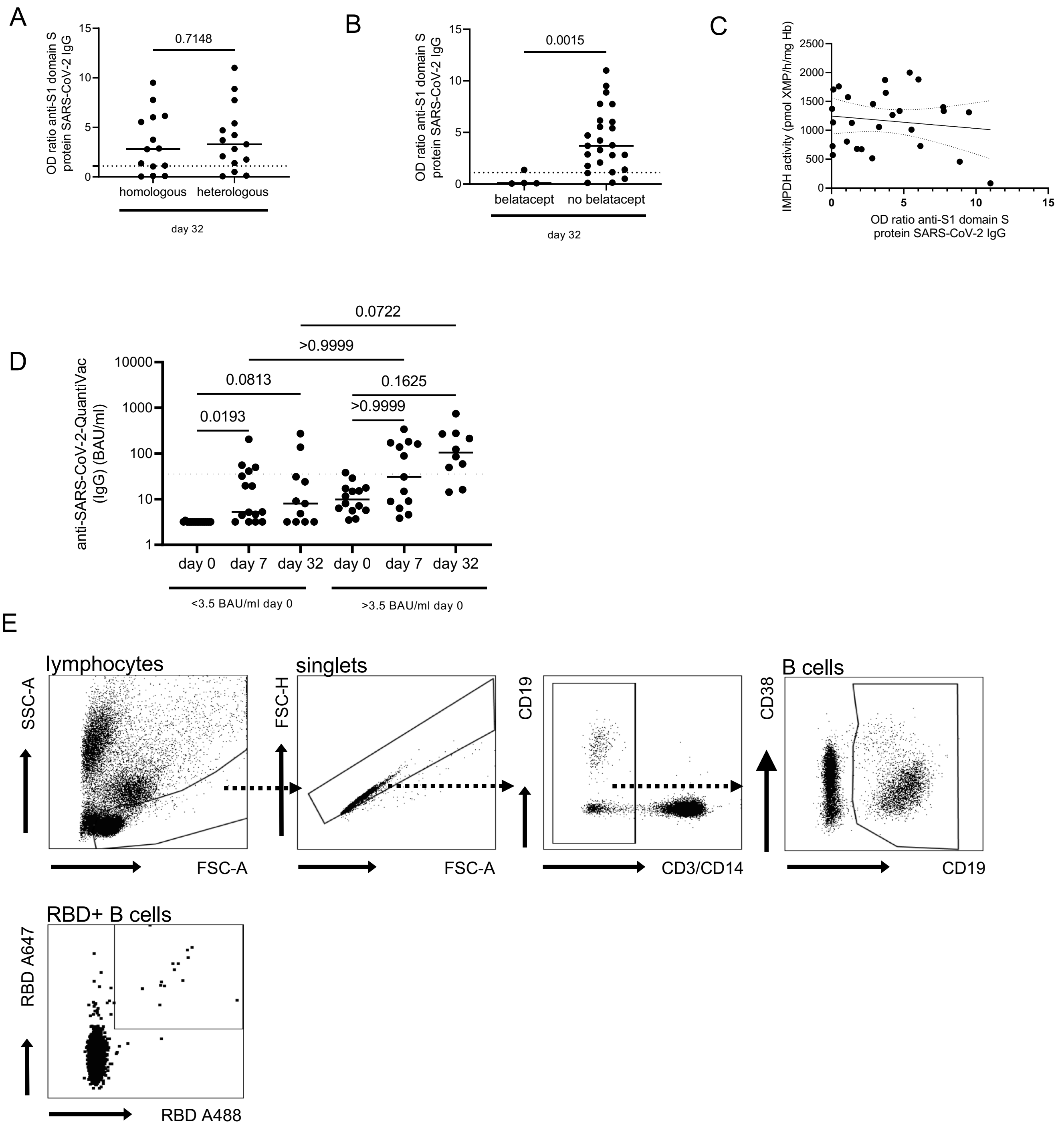

(A) Humoral vaccine-specific immune responses were assessed by ELISA for anti-spike protein S1 IgG and compared in patients with a homologous versus a heterologous previous vaccine protocol on day 32 after 4th vaccination. (B) Humoral vaccine-specific immune responses were assessed by ELISA for anti-spike protein S1 IgG and compared in patients with vs. without belatacept based immunosuppression on day 32 after 4th vaccination. The dotted lines mark the threshold for positivity (OD ratio of 1.1). (C) Correlation of inosine monophosphate dehydrogenase (IMPDH) activity in erythrocytes and anti-spike protein S1 IgG on day 32 after 4th vaccination. (D) Comparison of complete non-responders defined as IgG <3.5 BAU/ml (n=15) and partial non-responders IgG >3.5 BAU/ml (n=14) according to QuantiVac before 4th vaccination (complete cohort see Figure 1D) (E) Detection of SARS-CoV2 vaccine specific B cells. B cells in PBMCs were detect by flow cytometry. Antigen-specific B cells were identified by double staining with recombinant purified RBD conjugated to AF647 or AF488, respectively.

Supplemental Figure 2, Schrezenmeier et al.

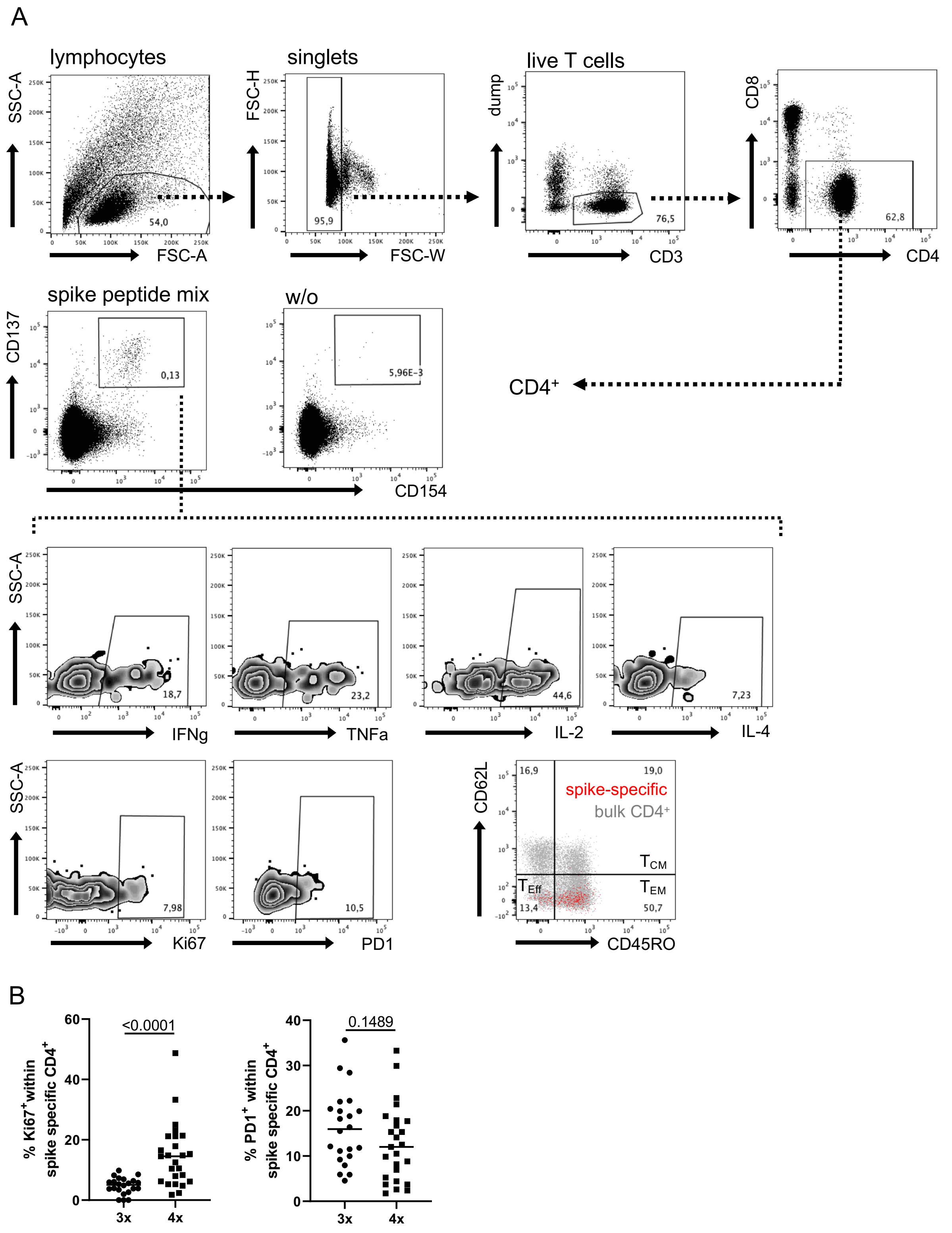

Detection of SARS-CoV-2 vaccine specific T helper cells. (A) Human PBMC were stimulated with SARS-CoV-2 spike peptide mix or left unstimulated for 16 h. Vaccine-specific live single CD14$^-$CD19$^-$CD3$^+$ ("dump" negative) CD4$^+$ T helper cells were detected by flow cytometry based on co-expression of CD154 and CD137. Specific cells were subsequently analyzed for expression of IFNγ, TNFα, IL-2 and/or IL-4, for the in vivo induced proliferation/activation markers Ki67 and PD1, or for their memory phenotype based on CD45RO and CD62L expression (T_{CM}: central memory-, T_{EM}: effector memory-, T_{eff}: effector T cells). Gates were set according to the respective unstimulated or unstained controls. (B) Comparison of Ki67 and PD1 expression in spike specific CD4$^+$ T cells 1 week after the 3rd SARS-CoV2 dose under standard IS therapy (separate cohort) vs. 1 week after the 4th dose under mycophenolate hold (cohort that is examined in this publication throughout).
