# Supplementary material for: Temporary antimetabolite treatment hold boosts SARS-CoV-2 vaccination–specific humoral and cellular immunity in kidney transplant recipients
Source: JCI Insight. 2022 May 9;7(9):e157836. doi: 10.1172/jci.insight.157836 (PMC9090237; doi:10.1172/jci.insight.157836)
Supplement: Supplemental table 1 [file jciinsight-7-157836-s061.pdf]

**Supplementary Table 1: Patient demographics of comparative cohort (Schrezenmeier et.al.)(1)**

**Table I**

Characteristics of kidney transplant (KTX) patients enrolled (n=25)

| **Variable** | |
|---|---|
| Age (mean yrs ± SD) | 59.7 (13.8) |
| Females (%) | 11 (44.0) |
| Caucasians (%) | 25 (100) |
| Heterologous vaccine protocol | 11 |
| **Clinical Parameters** | |
| Time since Tx (mean yrs ± SD) | 10.4 (8.69) |
| Retransplantation (%) | 5 (20.0) |
| Acute graft rejection (%)[A] | 0 (0) |
| IS medication | |
| CS+Tac+MMF (%) | 13 (56.0) |
| CS+CyA+MMF (%) | 7 (28.0) |
| $mTOR_i$+MMF±CS (%) | 3 (12.0) |
| CyA+$mTOR_i$ (%) | 1 (4.0) |
| CS+MMF (%) | 1 (4.0) |
| Comorbidities | |
| Hypertension (%) | 23 (92.0) |
| Coronary heart disease (%) | 6 (24.0) |
| History of myocardial infarction (%) | 2 (8.0) |
| Diabetes (%) | 5 (20.0) |
| History of liver disease (%) | 4 16.0) |
| History of malignancy (%) | 6 (24.0) |

CS – Corticosteroids, Tac – Tacrolimus, CyA – Cyclosporin A, MMF – Mycophenolate Mofetil, $mTOR_i$ – mammalian target of rapamycin inhibitor. SD – standard deviation. [A] until 19-27 days after third vaccination

1. Schrezenmeier E, Rincon-Arevalo H, Stefanski AL, Potekhin A, Staub-Hohenbleicher H, Choi M, et al. B and T Cell Responses after a Third Dose of SARS-CoV-2 Vaccine in Kidney Transplant Recipients. *J Am Soc Nephrol.* 2021.
